# Self‐incompatibility limits sexual reproduction rather than environmental conditions in an invasive water primrose

**DOI:** 10.1002/pei3.10042

**Published:** 2021-03-29

**Authors:** Luis O. Portillo Lemus, Michel Bozec, Marilyne Harang, Julie Coudreuse, Jacques Haury, Solenn Stoeckel, Dominique Barloy

**Affiliations:** ^1^ ESE, Ecology and Ecosystem Health Institut Agro INRAE Rennes France; ^2^ IGEPP INRAE Institut Agro Univ Rennes Le Rheu France

**Keywords:** heteromorphic self‐incompatible system, freshwater invasion, climate impact, management plan, *Ludwigia grandiflora* subsp. *Hexapetala*

## Abstract

Fruit‐set and seed‐set depend on environmental conditions and reproductive systems. They are important components of sexual reproductive success in plants. They also control the ecological success and adaptation of invasive plants within their non‐native ecosystems. We studied which factors bring about fruit‐set and seed‐set in invasive populations of the aquatic plant *Ludwigia grandiflora* subsp. *hexapetala*. We analyzed fruit set and seed set in 37 populations growing under variable climatic conditions in Western Europe. Sub‐samples of seven fruitful and fruitless populations were grown in common controlled conditions. We carried out self‐ and cross‐pollinations, and measured the floral morphometry. Environmental conditions did not affect fruit‐set and seed‐set in‐situ and in common controlled environments. Hand‐pollinations showed that individuals from fruitful populations exhibited fruit and seed production whatever the pollen donor, whereas individuals from fruitless populations only did so when pollen came from fruitful populations. Floral morphometry evidenced the existence of two floral morphs that fully overlapped with fruitfulness, and individual incompatibility. Our results rebutted the hypothesis that environmental variations control fruit set and seed set in these invasive populations. We instead showed that fruit set and seed set were controlled by a heteromorphic reproductive system involving a self‐incompatible and inter‐morph compatible morph (long‐styled morph), and a self‐ and inter‐morph compatible reverse morph (short‐styled morph). We collected morphs and fruit set records of this species worldwide and found the same relationship: fruitless populations were all composed only of individuals with long‐styled floral morph. Our study constitutes the first evidence of a heteromorphic self‐incompatible system in Ludwigia genus and Onagraceae family.

## 1 INTRODUCTION

Reproductive success is a central biological feature key to understanding the ecology and evolution of populations and species, and for managing endangered, invasive, cultivated, or unwanted populations (Barrett et al., 2008). In Angiosperms, the first two decisive steps of sexual reproductive success require that both the individual and population produce fruit from their flowers through successful pollination and viable seeds from their gametes, which are then able to germinate to give the next generation (Obeso, 2002). Fruit‐set and seed‐set are essential resources for ecosystems and human activities, and efficient proxies in quantifying the sexual reproductive success of individuals and populations (Sutherland, 1986). Therefore, identifying environmental and biological processes that drive fruit‐set and seed‐set in plant populations is crucial to understand their ecology and evolution and manage their populations (Barrett, 1998; Sutherland, 1986).

Water primrose, *Ludwigia grandiflora* subsp. *hexapetala* (Hook. & Arn.) Nesom and Kartesz (2000), is one of the most aggressive aquatic invasive plants in the world (Thiébaut & Dutartre, 2009; Thouvenot et al., 2013). In recent decades, this species has been reported as invading freshwater ecosystems in 15 countries worldwide, threatening local biodiversity, and water accessibility for human activities (EPPO, 2011; Hieda et al., 2020). *Ludwigia grandiflora* subsp. *hexapetala* populations reproduce using vegetative fragmentation and sexual seeds. Although its vegetative growth is particularly well documented, the role and importance of sexual reproduction within the population are still unknown, in particular concerning how it contributes to the species invasiveness in newly colonized areas (Dandelot, 2004; Ruaux et al., 2009; Thouvenot et al., 2013).

Fruit and seed‐sets are known to be affected by abiotic environmental factors, such as sunshine, temperature and hygrometry, and biotic factors, including abundance of pollinators for entomophilous plants, flower grazers, and seed diseases (Giles et al., 2006; Grass et al., 2018; Harder & Aizen, 2010; McCall & Irwin, 2006; Sun et al., 2018; Sutherland, 1986). These environmental factors are thus key to understanding the sexual reproduction of plants invading new areas, especially in the context of global changes. However, fruit‐set and seed‐set can also be affected by internal mechanisms developed by plants in order to avoid self‐pollination (Barrett, 1998; Dellaporta’ & Calderon‐Urrea, 1993; Sutherland, 1986). The majority of flowering species mate using self‐incompatibility (SI) systems, which result in the inability of individuals to produce zygotes with self‐pollen (De Nettancourt, 2001). Self‐incompatible species present lower or even no fruit set and seed set when populations lack compatible partners (Brys & Jacquemyn, 2020; Sutherland, 1986; Sutherland & Delph, 1984).

Within SI systems, heteromorphic SI systems include all plants with a physiology limiting self‐pollination associated with either two (distyly) or three (tristyly) different morphs of hermaphroditic flowers with a spatial separation of their styles and anthers, that is, herkogamous flowers (Barrett, 2019). Heteromorphic self‐incompatibility, also known as intra‐morph incompatibility, prevents self‐ and intra‐morph pollinations (Barrett & Cruzan, 1994). Distylous species genetically express two types of floral morphs, differing in their reciprocal heights of styles and stamen (reciprocal herkogamy). Currently, distyly, the most‐common heterostylous system, has been described in 25 families, among which are *Polygonaceae*, *Menyanthaceae*, and *Turneraceae* (Barrett, 1998). Although these previous morphological features are valid for most heterostylous plants, several species show deviation from standard reciprocal herkogamy and morphologic compatibility patterns (Barrett, 1998). For example, *Narcissus assoanus* and *Jasminum malabaricum* present non‐reciprocal herkogamy, with only the style height dimorphism, while their stamens remain in the same position in both morphs (Cesaro & Thompson, 2004; Ganguly & Barua, 2020).

The Onagraceae family includes about 657 species of herbs, shrubs, and trees in 17 genera (Les, 2017; Munz, 1942; Wagner et al., 2007; Zardini et al., 1991). Figure S1 illustrates the wide floral diversity found in the *Onagraceae* family. In this large family only the homomorphic gametophytic self‐incompatibility (GSI) system has been described to date, based on just two species: *Oenothera organensis* and *Oenothera rhombipetala* (Gibbs, 2014). Within the Onagraceae, the *Ludwigia* genus includes 83 species, of which 75 species were classified as generally self‐compatible with seven self‐incompatible species (Zardini & Raven, 1992). *Ludwigia* section *Oligospermum* comprises a group of nine highly variable species, including our study model, *Ludwigia grandiflora* subsp. *hexapetala*, and have similar morphology features such as 5(6)‐merous flowers. *Ludwigia hexapetala* (Zardini et al., 1991) and *Ludwigia grandiflora* subsp. *hexapetala* (Nesom & Kartesz, 2000) are synonymous scientific names. *L*. *grandiflora* subsp. *hexapetala* is the species name officially used in the European Union's (UE) legislation concerning invasive species (EPPO, 2011) and that sounds for invasive plant managers. We hereafter referred to as *Lgh*.

Invasive populations of *Lgh* in France present contrasting fruit‐set and seed‐set depending on their geographical areas. On the Atlantic side of Europe, *Lgh* populations produced fruits and viable seeds while in the Mediterranean zone, all *Lgh* populations remained fruitless (Dandelot, 2004). Although, all those European populations massively bloomed with the presence of a multitude of insect foraging their flowers. Those observations led scientists and environmental managers to conclude that sexual reproductive success in invasive populations depends on climatic factors, and to organize plan control accordingly (Dandelot et al., 2005). However, invasive populations of *Lgh* were described as having stigmas above the anthers on flowers with either five or six component parts in a distinct whorl of a plant structure (merosity; Dandelot, 2004).

Here, we aimed to understand if observing fruitful and fruitless populations can be explained either by environmental conditions as previously proposed (Dandelot, 2004) or due to a self‐incompatible system that we newly characterized in the present paper. We thus first tackled if and when environmental factors affected fruit set and seed set in Western Europe populations. Then, we made hand‐controlled pollinations in greenhouse experiments allowed us to characterize the mating system of *Lgh*. Since *Lgh* showed varied floral morphology, we analyzed the floral morphometry to characterize the floral morphs. Our results i/ contributed to qualify and understand the mating system of *Ludwigia* genus, ii/ revealed a new type of mating system not yet described in the Onagraceae family, and iii/ contributed to show that we should not rely on natural environmental variations to limit the invasibility of this species worldwide as previously assumed.

## 2 MATERIALS AND METHODS

### 2.1 Plant development

Vegetative growth of *Lgh* starts in April with the production of submerged foliage stems, then each stem produces a single flower every 3 days. Anthesis occurs early in the morning, and bees, beetles, and flies actively pollinate flowers (Dandelot, 2004). The fruit (capsule) needs 6 weeks after pollination to become mature. After pollination, fruit develops from August to November, and the aerial parts of the plants degenerate in late autumn. Populations are perennial in the invaded areas as in their native range (Dandelot, 2004; EPPO, 2011).

### 2.2 Fruit‐set, seed‐set, and climatic variations in field populations

**FIGURE 1:**
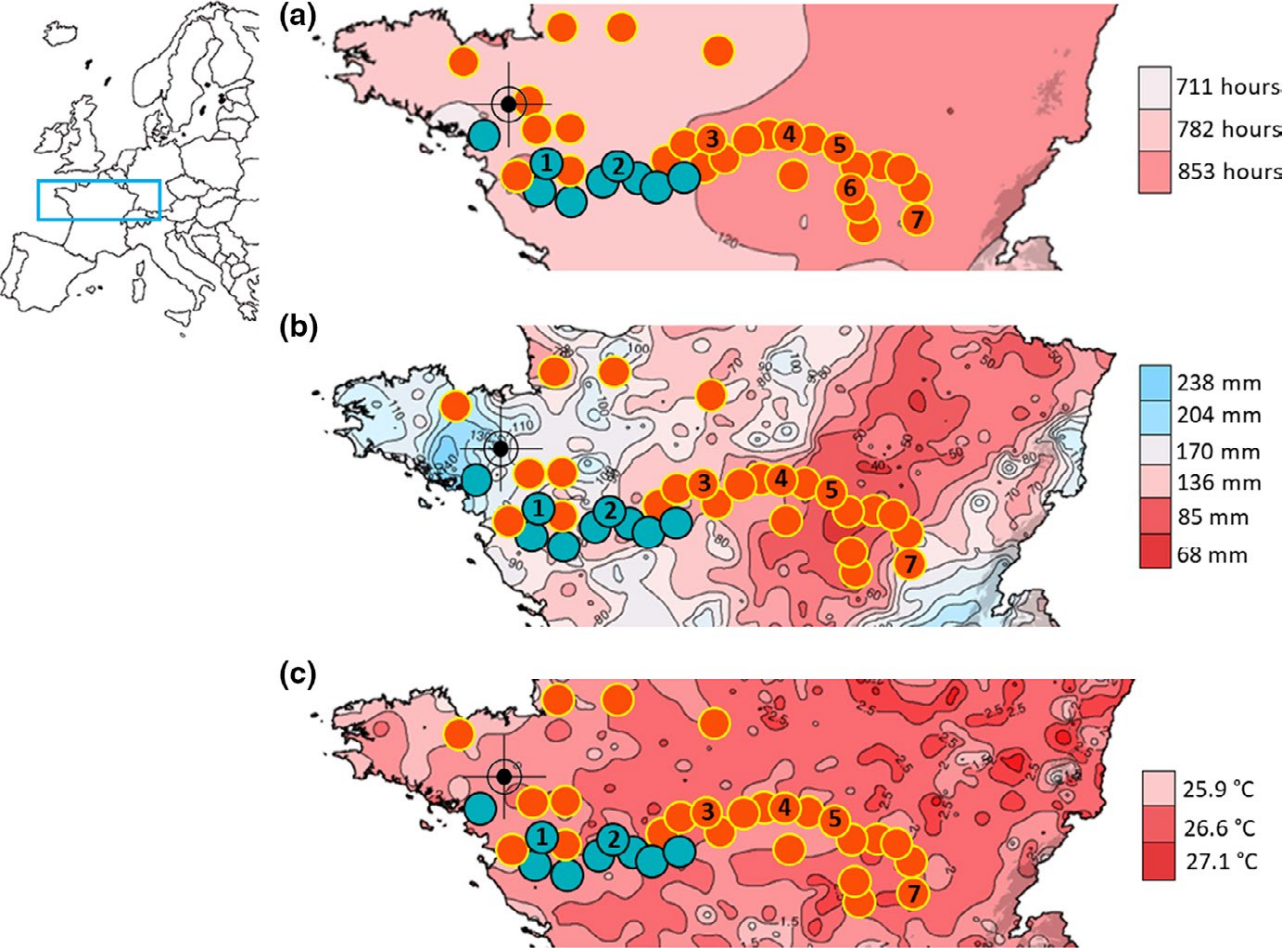
Location of nine studied fruitful (blue circles) and 28 fruitless (orange circles) populations mapped with climatic conditions. Number in circles indicates the geographical positions of the seven sampled populations: 1, Maze (Mazerolles); 2, Pont (Ponts‐de‐Cé); 3, Orl (Orléans); 4, Poui (Pouilly‐sur‐Loire); 5, Gill (Gilly‐sur‐Loire); 6, Chat (Châtel‐de‐Neuvre) and 7, Cham (Chambéon) populations. The dark target symbol locates our common garden. See support information Table S1 for GPS Locations. (a) heat‐map of cumulative sunshine hours in summer; (b) heat‐map of cumulative millimeter of precipitation in summer; and (c) heat‐map of the mean summer temperatures. All three maps were generated from the meteorological databases of “météo France”

We focused our study on populations invading Western Europe along a West–East transect in France in the Loire river watershed (Figure 1). The Loire receives the waters of one of the largest drainage basins in Western Europe and is the longest French river (Vogt et al., 2007). Its 117,500 km^2^ are known to cover a wide climatic gradient, from oceanic to continental, including variations in sunshine, temperature, and precipitation. Invading populations in this area were previously mentioned to be fruitless in all varied environments, however, some were recently reported to be newly producing fruit (Haury et al., 2012). In this watershed, we monitored the fruit sets and seed sets of 37 populations in situ along 765 km of the Loire River, its main tributaries and a few surrounding ponds (Table S1).

In early October 2018, we measured fruit set in the 37 in situ populations as the mean of the raw quantity of fruit produced per stem from 40 measured stems. The stems were randomly picked by groups of eight stems within five 1 m^2^ squares set along a 40 m transect, separated by an interval of 10 m. For two in situ fruitful populations (Mazerolles and Ponts‐de‐Cé), we also measured seed set as the ability of fruit to produce seeds that germinated successfully, and resulted in viable plants. We randomly sampled five ripe fruits per population in which we counted the quantity of seeds, and quantified the number of living plants obtained 1 month after seed germination.

To assess whether climatic conditions impacted fruit set, we compared the distributions of climatic variations between fruitful and fruitless populations (Figure 1; Table S1). In the nine fruitful populations, we correlated the quantity of seeds and the number of living plants obtained 1 month after seed germination with the climatic variations. For each population location, climatic variations, that is, sunshine, temperature, and precipitation data, were compiled from *Meteo France database* over the flowering time from June to August. We averaged climatic data recorded every hour over the last 20 years (http://www.meteofrance.fr/climat‐passe‐et‐futur/bilans‐climatiques).

### 2.3 Fruit set, seed set, and seed viability in a common garden

In early June 2018, we sampled seven of the 37 field populations in order to populate a common garden, and a greenhouse, and study their fruit set and seed set in controlled conditions (location: Agrocampus Ouest, Rennes, France. 48°06′47.7″N 1°42′30.2″W).

The common garden ensured the same environmental and climatic conditions for all individuals sampled from different populations. If fruit set and seed set were controlled by environmental conditions, a common garden should homogenize the reproductive success of all individuals whatever their previous in situ measures. To populate the common garden, we randomly sampled 40 stems per in situ population, extracted from five squares of 1 m^2^ at an interval of 10 m on a linear transect, in which we randomly chose eight stems. All 40 stems of a same population were installed together in a 450 L mesocosm. A total of 280 sampled plants distributed in seven mesocosms were installed in the common garden. Each mesocosm was isolated with an insect‐proof net, thereby only allowing intra‐population pollination (hereafter named the ‘free pollination’ treatment). To ensure pollen transport between and within flowers in free pollination treatments, we supplied each mesocosm with ~350 pollinating flies (*Calliphora erythrocephala*) every 3 weeks during the flowering period.

In experimental populations, we measured fruit set as the quantity of fruit produced by each stem divided by its recorded number of flowers, counted and tagged by a wool yarn every 3 days. In each mesocosm, we measured the fruit set on 40 stems in the summer of 2018 (during the full flowering season) then on 40 other stems at the beginning of October 2018 (at the end of the flowering season). We also measured seed set by sampling five ripe fruits per mesocosm from the fruit set survey, counting all their seeds, and then the consequent number of surviving plants obtained 1 month after germination, as we did for the in situ fruitful populations.

We previously noticed that success of seed germination in *Lgh* required a seed dormancy interruption, but no vernalization, that is, floral induction by cold. To assess germination success, we thus used a modified Hussner et al. (2016) germination method: we put the fruits in water in Petri dishes at 4°C for a minimum of 3 weeks (cold‐stratification period). We then deposited the seeds in basins in soil saturated with water at a temperature of 25°C and a photoperiod of 16:8. Seeds began to germinate after 4 to 7 days.

### 2.4 Fruit set, seed set, and seed viability at different controlled temperatures

Greenhouse experimentations, beyond ensuring the same environmental conditions for all individuals sampled from different populations, enabled us to control temperatures in order to test their effects on fruit‐set. We randomly selected 10 individuals per mesocosm that were then cloned by cutting, and installed together in 80 L containers as replicates. This subsample of 70 plants was distributed into seven containers (one per sampled population), and installed in a greenhouse in early June 2018, allowing us to manage the temperature (Schema of sampling protocol in Figure S2).

In the greenhouse, we only measured fruit set on the hand‐controlled pollinated flowers in each container. Three days after hand‐controlled pollination, we counted the number of aborted flowers, and the number of fruits in formation. All fruits produced through controlled pollination were harvested at full maturity in order to assess their seed set. Fruit set was measured on 15 flowers and seed set on three ripe fruits per fruitful hand‐pollination.

We assessed the sexual compatibility of individuals at controlled temperatures by conducting a full scheme of hand‐controlled pollinations between and within populations growing in the greenhouse, and then measuring their fruit set and seed set. We carried out two types of hand‐controlled pollination: intra‐individual pollination (self‐pollination) and inter‐individual pollinations, that is, cross‐pollination between individuals from the same population (named intra‐population controlled cross) or from another population (named inter‐population controlled cross). When flower buds appeared, we locked them in cellophane bags to protect the flowers from incoming pollen. To ensure self‐pollination, we shook the flowers in the bags after anthesis, and visually checked that pollen was deposited on the stigma. For inter‐individual pollination, in order to simulate free random crosses, we selected five pollen‐donor flowers and five other flowers which were to receive the selected pollen on their pistil. The pistil‐donating flowers were emasculated before anthesis, and then pollinated with a mix of pollen from the five pollen‐donating flowers. After pollination, the five pollinated flowers were sealed in cellophane bags to protect them from uncontrolled pollen incomings.

**FIGURE 2:**
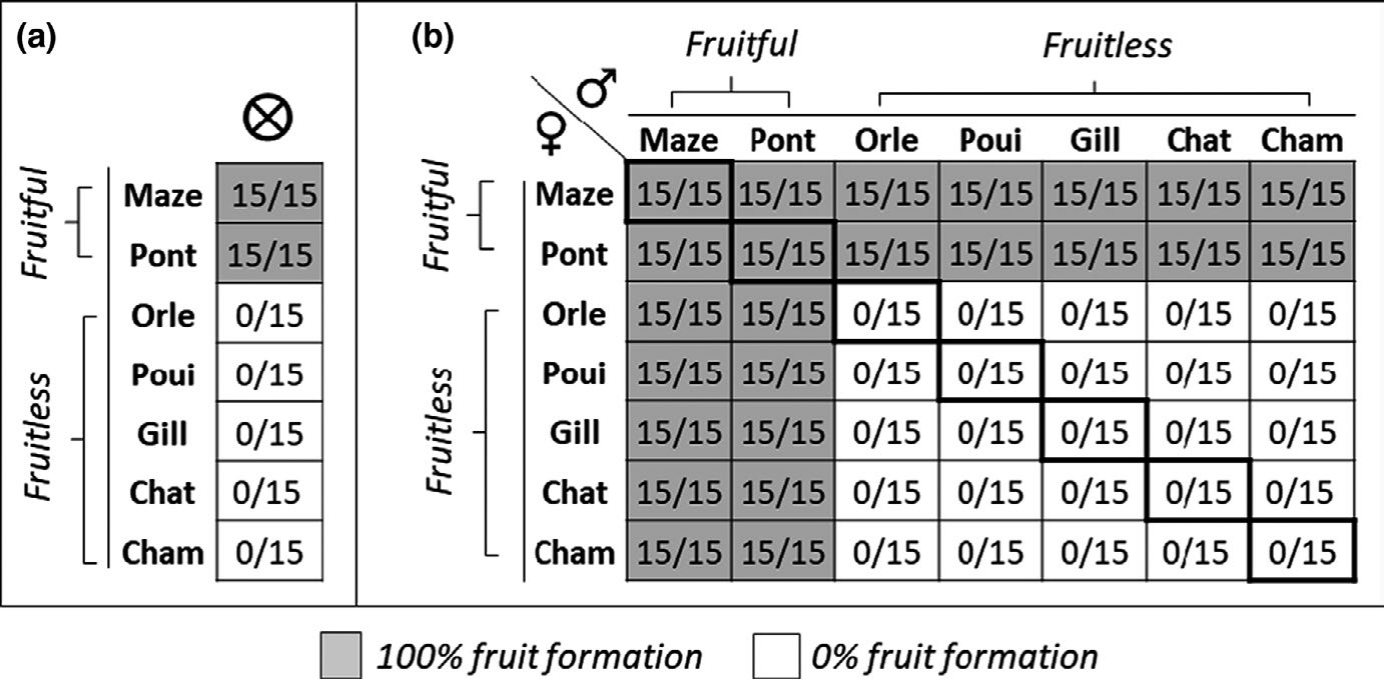
Fruit set in the seven sampled populations of *Ludwigia grandiflora* subsp. *hexapetala* after hand‐controlled‐pollination crosses from mid‐July to beginning of August 2018 in greenhouse. (a) Results of self‐pollinations. (b) Results of intra‐population (diagonal line) and inter‐population (other boxes) hand‐controlled cross‐pollinations. Numbers separated by a slash indicate the ratio between fruits obtained from a fixed—15—number of flowers. A total of 840 pollinated flowers were hand‐controlled pollinated: 105 self‐pollinated flowers, 105 intra‐population cross‐pollinated flowers, and 630 inter‐population cross‐pollinated flowers, corresponding to 15 flowers per population per pollination condition. Similar hand‐controlled crosses matrix made in May to August 2019 in greenhouse under six different temperatures 22°C, 27°C, 30°C, 35°C, 40°C, and 45°C. Five flowers per population per condition and per temperature were self‐, intra‐, and inter‐population pollinated. The same results were obtained

From mid‐July to early August 2018, we quantified the fruit set of individuals of (i) 105 self‐pollinations, (ii) 105 intra‐population pollinations, (iii) 630 inter‐population pollination crosses between individuals from different populations. Indeed, crosses of the seven focused populations resulted in 42 inter‐pop combinations. For example, we performed 15 cross‐pollinations with Maze‐♀ × Pont‐♂ and 15 cross‐pollinations with Maze‐♂ × Pont‐♀ (Figure 2). In January 2019, we quantified the seed set of three fruits obtained from each fruitful cross.

We performed the same replicates of self‐, intra‐population, and inter‐population hand‐controlled pollinations every 15 days from May to August 2019 at seven increasing temperatures (18°C, 22°C, 27°C, 30°C, 35°C, 40°C, and 45°C, each varying by a max. +/−2°Cover a full day) to measure its impacts on fruit set and seed set within and between the seven sampled populations. At each temperature, we carried out five self‐pollinations and five intra‐population cross‐pollinations per population; and five inter‐population cross‐pollinations for each of the 42 inter‐population combinations.

From May to October in 2018 and 2019, we watered both the common garden mesocosms and greenhouse containers every 15 days with a commercial nutrient solution (composed of 6%N, 6%K, 6%P) during the growth and flowering periods, to ensure all plants were growing without nutritional deficiencies.

### 2.5 Floral morphology in *Ludwigia grandiflora* subsp. *hexapetala* and implication on experimental and field fruit set and seed set

We analyzed fruit set in relation to individual and population floral morphology. Within species and populations, flowers could vary in their numbers of component parts in a distinct whorl of a plant structure, named merosity. To assess variation in floral morphology of *Lgh* within populations, we recorded the merosity of 480 flowers in each mesocosm of the common garden, sampled from mid‐July to early August 2018, and quantified the frequency distribution of merosity per population. On a random subset of 60 total flowers, we measured 10 floral morphological traits. Measured traits were the length and width of the sepal and petal, length of the stamen and anther for the first and the second whorls, length of the pistil and width of the floral receptacle, and production of nectar (Table S2). We measured these floral traits with a digital caliper (0.01 mm accuracy), except for styles. We estimated nectar production by sampling the nectar produced by five flowers per population using a graduated 10µL micropipette. To evaluate floral morphology, we measured all the morphological traits (except for the pistil) of 30 flowers sampled on individuals from fruitless populations (six flowers per population × five fruitless populations), and 30 flowers sampled from individuals from fruitful populations (15 flowers per population × two fruitful populations). We measured 150 styles from 75 flowers from both fruitful populations, and fruitless populations.

### 2.6 Fruit set in other worldwide native and invasive populations

To assess the generality of our findings concerning the impacts of the environment and the mating system on invasive water primrose in the Loire watershed, we collected and analyzed web data (from sourced photographs, herbaria, papers, wildlife services, and surveys) on populations in native and invasive areas, to which we associated floral morphs (using our own floral morphometry criteria) with their reported fruit and seed productions (Table S3). Hereafter, we named populations in which stems produced fruits as fruitful, and populations with no fruit as fruitless.

### 2.7 Statistical analysis

Fruit set, seed set, germination, climate, and morphometry measures were not normally distributed, and we cannot ensure the homogeneity of their variances in all analyses. We thus analyzed our data using non‐parametric tests.

To assess whether fruitfulness (if the population produced fruit or not, Bernoulli‐type measure) in the field, common garden, and greenhouse populations varied with environmental conditions (mean temperature during the flowering season, cumulative rain fall, and cumulative sunshine hours), we used a Kruskall–Wallis test (test non‐parametric equivalent of ANOVA; Kruskal & Wallis, 1952), with the null hypothesis that all groups shared the same median. For each test, we provided the mean ±standard deviation of the climatic variations in both fruitful and fruitless populations.

To assess whether climate, fruit set, seed set, and germination measures (positive continuous measures) were correlated, we used a Kendall partial rank‐order correlation (non‐parametric equivalent of Pearson correlation coefficient; Kendall, 1938).

To assess whether categorical variables such as populations or treatments resulted in different fruitfulness, fruit set, seed set, and germination, we used a Kruskall–Wallis test with the null hypothesis that all groups shared the same median. When post‐hoc analyses were needed, we used a Conover–Iman multiple comparison approach using the pairwise rank sums between population or treatment categories (Conover & Iman, 1979).

To assess whether merosity followed the same distributions in fruitful and fruitless populations, we performed a classic Chi‐square test of independence for merosity frequencies in a contingency table. To assess the number of floral morphotypes in populations, we applied an unsupervised k‐means clustering (Forgy, 1965; Lloyd, 1982) on floral morphometry measures and kept the best fit model as the number of clusters that maximized the curvature on the sum of squared distance (SSE) between measures and assigned cluster centroids, exploring an increasing number of clusters from one to 15 (classical Elbow method). We assessed which morphometric measures varied between floral morphs using a Kruskall–Wallis test with the null hypothesis that all floral groups shared the same median measure. We also explored how morphometric measures covariated with identified floral morphs using results obtained from a principal component analysis (PCA) on floral morphometry.

Finally, we tested whether one floral morph prevailed in worldwide invasive populations by computing the cumulative distribution function to test if one floral morph increased its frequency in invasive ranges compared to those found in its native area.

Kruskall–Wallis tests, Conover–Iman post‐hoc analyses, Kendal partial rank‐order correlation, and the Chi‐square test of distribution independence were computed using *Scipy 1.6.0* (Virtanen et al., 2020). Unsupervised k‐means clustering on floral morphometry was achieved using *Scikit‐Learn 0.24* (Pedregosa et al., 2011) and its associated *Kneed 0.6.0* elbow algorithm. PCA were computed from the ade4 package in *R* (Thioulouse et al., 2018).

## 3 RESULTS

### 3.1 Fruitfulness in field invasive populations of *Ludwigia grandiflora* subsp. *hexapetala* in Western Europe

All 37 monitored populations in the Loire basin massively blossomed from June to August. However, around 76% of the populations (28/37) were fruitless (Table S1). We mainly found fruitful populations on the western ocean side, and fruitless populations on the eastern continental side (Figure 1; Table S1). The western area encompassed both fruitful and fruitless populations (including the two fruitful populations sampled in order to populate the common garden, Maze and Pont), while the eastern area only showed fruitless populations (including the five fruitless populations sampled to populate the common garden, Orl, Poui, Gill, Chat, and Cham, Figure 1).

### 3.2 Environmental implication on fruit set and seed set

Climatic data showed variations between studied populations along the watershed (Figure 1). In the 37 monitored populations, cumulative sunshine hours ranged from 711 to 853 h (Figure 1a), pluviometry ranged from 86 to 204 mm (Figure 1b) and mean summer temperatures only ranged from 25.9 to 27.1°C (Figure 1c). Fruitful populations showed higher mean summer temperatures (fruitful populations: 25.4 ± 1.2°C, fruitless: 24.8 ± 1.4°C, *H* = 11.79, *p* < 10^−3^), higher cumulative summer rainfall (fruitful populations: 58.2 ± 9.1 mm, fruitless: 49.7 ± 5.2 mm, *H* = 30.89, *p* < 10^−7^) and slightly higher cumulative summer sunshine hours (fruitful populations: 231.2 ± 18.7H, fruitless: 227.7 ± 13.3H, *H* = 3.30, *p* = 0.069). Within in situ populations, climatic variations were all strongly correlated (*r*_τ_ = 1.0, *p* < 10^−13^). Fruitful populations did not vary in their production of fruits (*H* = 5.56, *p* = 0.696), seeds per stem (*H* = 1.71, *p* = 0.989), total number of seeds of 40 stems (*H* = 5.85, *p* = 0.664), and number of viable seeds that successfully germinated (*H* = 12.03, *p* = 0.150). Therefore, fruit set, seed set per stem, total seed set of 40 stems, and number of viable seeds that germinated did not varied with mean summer temperatures, cumulative summer rainfall, or cumulative summer sunshine hours (*r*_τ_[−0.7,0.16], *p* > 0.158).

**FIGURE 3:**
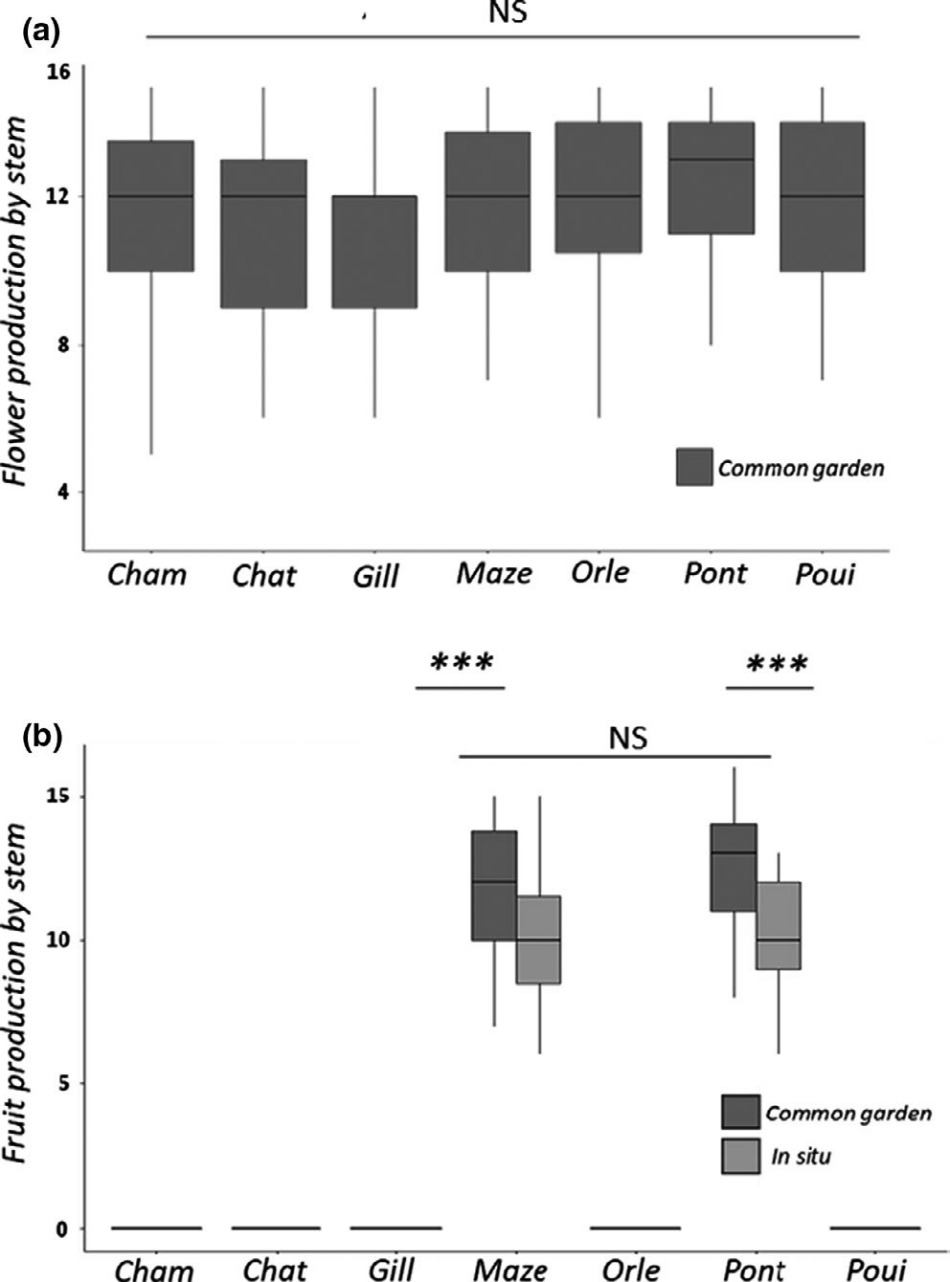
Distributions of flower (a) and fruit (b) productions per stem and population of *Ludwigia grandiflora* subsp *hexapetala* obtained in the common garden (dark gray boxes) and in situ (gray boxes) from July to early October 2018. A total of 40 stems were observed by populations in situ and in common garden conditions. (a) All experimental populations produced the same quantity of flowers in the common garden (*H* = 7.21, *p* = 0.302). (b) In both in situ and the common garden, we observed two types of population: fruitless and fruitful. Maze and Pont were fruitful in both common garden and in situ conditions. The five populations Cham, Chat, Gill, Orle, and Poui in both common garden and in situ conditions were fruitless. The two fruitful populations produced the same quantity of fruits (*H* = 38.47, *p* < 10^−7^) both in situ (*U* = 915, *p* = 0.26) and in common garden (*U* = 649, *p* = 0.14, even though both of their fruit productions were higher in common garden conditions (common garden: median of 12.5 fruits per stem, in situ: median of 10 fruits per stem, Maze: *U* = 1172, *p* < 10^−3^; Pont: *U* = 1303, *p* < 10^−5^)

Populations chosen for populating the common garden covered the climatic variations measured along the Loire River. In the common garden, individuals sampled from the seven studied populations produced the same quantity of flowers per stem (*H* = 7.21, *p* = 0.302; Pairwise post‐hoc Conover p‐values all superior to 0.499): between six and 15 flowers per stem in all populations (Figure 3a). Despite the 40 individuals sampled from one geographical population being free to pollinate one another, and all populations sharing the same environmental conditions, only flowers from the two fruitful populations (Maze, Pont) again produced fruits: between six and 15 capsules per stem, with all their flowers eventually giving fruits (fruits–flowers ratio = 1, Figure 3b). Fruit production per stem was similar in Maze and Pont populations (*H* = 38.47, *p* < 10^−7^), whether in situ (*U* = 915, *p* = 0.26) or in common garden samples (*U* = 649, *p* = 0.14, Figure 3b). Samples from both fruitful populations, however, produced more fruit per stem in the common garden (median of 12.5 fruits per stem) than in situ (median of 10 fruits per stem, Maze: *U* = 1172, *p* < 10^−3^; Pont: *U* = 1303, *p* < 10^−5^, Figure 3b).

All fruits obtained from fruitful in situ and common garden populations gave the same number of seeds, and the same total number of seeds per 40 stems (*H* = 1.14 *p* > 0.99 and *H* = 5.11, *p* > 0.64, respectively). Fruits produced from both fruitful populations contained similar seed set and plant production (Figure S3). Seed sets from Mazerolles and Pont‐de‐Cé showed similar germination rate (>93%) (Figure S3, ratio of plant production over seed set per fruit).

In the greenhouse, after self‐ and intra‐population controlled pollinations at seven different temperatures (18°C, 22°C, 27°C, 30°C, 35°C, 40°C, and 45°C), we obtained the same results as obtained in the common garden: again, at all temperatures, individuals sampled from fruitful populations all gave fruits and seeds, while all individuals sampled from fruitless populations remained fruitless and seedless.

### 3.3 Floral morphology in *Ludwigia grandiflora* subsp. *hexapetala*

We found that invasive populations showed variations in their floral morphologies. All populations produced 5‐, 6‐, and 7‐merous flowers (Figure S4), but merosity distributions differed among the seven focused and sampled populations (*χ* ^2^=28.6, *p* < 10^−6^). Both fruitful populations (Pont and Maze) respectively produced 70 and 80% 5‐merous flowers, 25 and 15% 6‐merous flowers, and less than 5% 7‐merous flowers’ (Figure S4a–c). Fruitless populations (Orl, Poui, Gill, Chat and Cham) showed higher proportions of 5‐merous flowers (90 to 95%), and less 6‐merous (4 to 9%) and 7‐merous flowers (less than 1%; Figure S4d–f).

**FIGURE 4:**
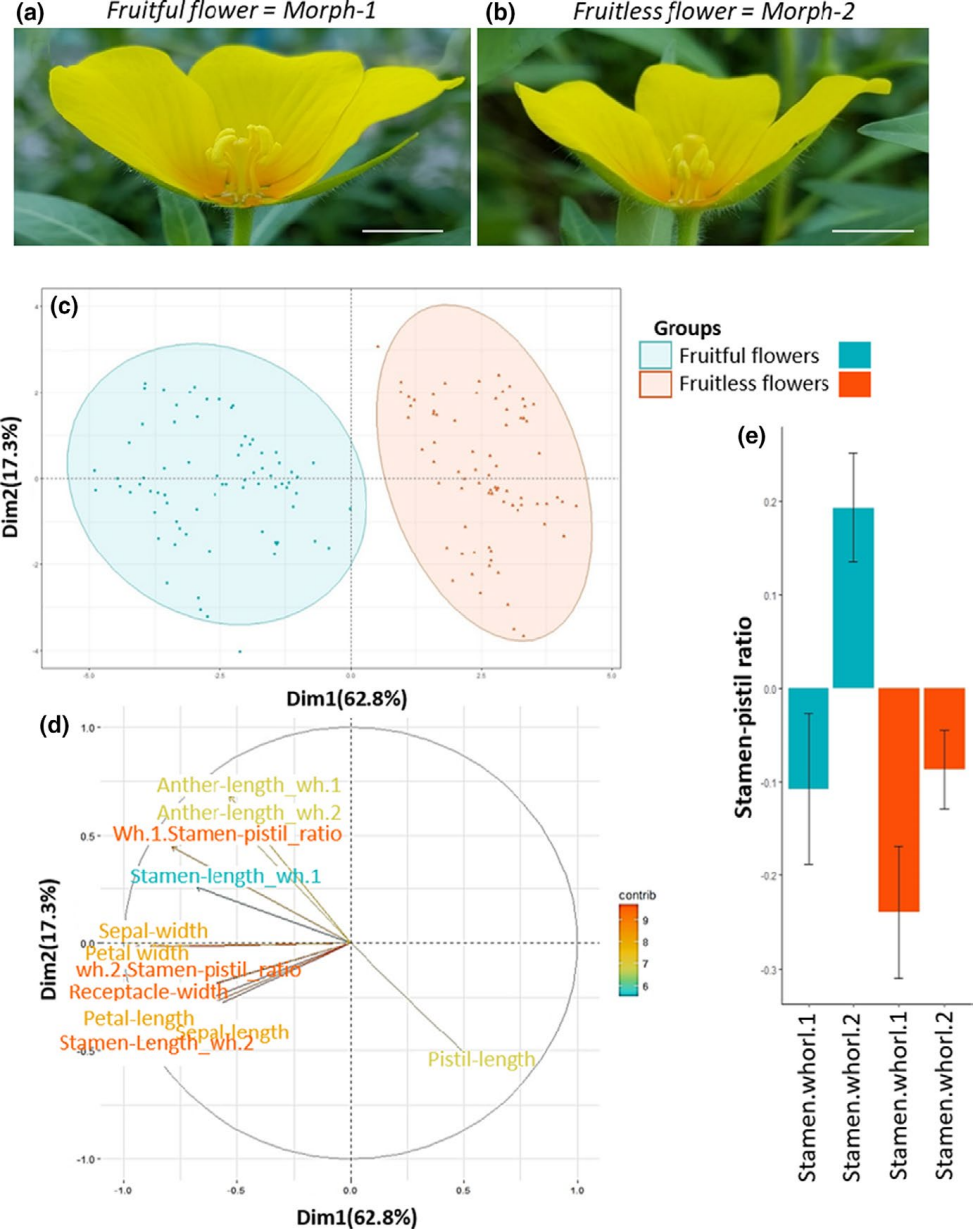
Floral morphometry of fruitful and fruitless populations of *Ludwigia grandiflora* subsp *hexapetala*. (a) Longitudinal sections of a typical flower from (a) fruitful populations and (b) from fruitless populations. Morphometry was measured from these sections. Bars = 1 cm. (c) Principal Component Analysis of floral morphometry: it showed two distinct clusters. These two floral groups fully coincided with fruitful (blue) and fruitless (red) groups. (d): Variable factor map of floral morphometry. Colors indicated the variable contributions from low (blue) to high (red). For a given flower having a larger, perianth, androecium, and floral receptacle showed a smaller pistil and vice versa. (wh.1 or wh.2 = whorl.1 or whorl.2) (e) Length stamen/pistil ratio of first and second whorls for fruitful (blue) and fruitless (red) flowers, bars represent standard deviations. In fruitful flowers, stamens from second whorl were always positioned above the pistil. In fruitless flowers, both stamen whorls were always positioned below the pistil

For the 5‐merous flowers, which were the most frequent merosity type found in both fruitless and fruitfull populations, our k‐means clustering analysis on floral morphometry measures found that the best fit model was obtained for two clusters (SSE for one group = 3600, for two groups = 1620.3, for three groups = 1347.6, for four groups = 1141.1, for five groups = 1020.0, and for six groups = 947.6). These two clusters, hereafter named morph‐1 and morph‐2, differed in flower sizes and all their measurements (*H* = 209, *p* < 10^−46^; Figure 4a,b). To visualize those two clusters, a PCA was realized (Figure 4c,d). Compared to morph‐2 flowers, morph‐1 flowers had larger and wider floral receptacles (Mean diameter: morph1 = 4.9 mm and morph2=3.7 mm), sepals (Mean length: morph1=18.3 mm and morph‐2 = 14.9 mm; Mean width: morph‐1 = 4.2 mm and morph‐2 = 3.2 mm), and petals (Mean length: morph1 = 27.2 mm and morph‐2 = 21.0 mm; Mean width: morph‐1 = 22.4 mm and morph‐2 = 16.3 mm; Table S2). Size ratio for all the floral pieces between morph‐1 over morph‐2 gave scaling factors of 1.3, 1.2, 1.3, 1.3, and 1.4, respectively. Morph‐1 flowers were consistently, 1.3 times the size of the morph‐2 flower for all of the floral measures. Interestingly, the pistil, stamen of the first whorl, and stamen of the second whorl showed a smaller scaling factor, of 0.9, 1.1, and 1.2, respectively. Moreover, morph‐1 whorl 2 stamens were above the pistil (i.e., short‐styled, reverse herkogamous, bigger flowers) while morph‐2 whorl 2 lower stamens were below the pistil (i.e., long‐styled, approach herkogamous, smaller flowers). Even though all measures significantly correlated with floral morphs and between them (*H* from 55 to 224, *p* < 10^−12^), the whorl‐2‐stamen‐pistil ratio was the most discriminating characteristic, and the most significant parameter for differentiating floral morph‐1 from morph‐2 (*H* = 224, *p* < 10^−50^; Figure 4e).

### 3.4 Floral morph implications on fruit set and seed set

In situ populations showed a perfect congruence between fruitfulness and floral morphs. Plants with morph‐1, short‐styled, reverse herkogamous, bigger flowers were only found in fruitful populations, while plants with morph‐2, long‐styled, approach herkogamous, smaller flowers were only found in fruitless populations. In the common garden, and thereby sharing the same environmental conditions, plants sampled from Maze and Pont all produced morph‐1 flowers as found in situ and were fruitful, while plants sampled from Orle, Poui, Gill, Chât, and Cham all produced morph‐2 flowers and were fruitless.

In the greenhouse, hand‐controlled self‐pollination and intra‐population pollination gave the same results obtained in the common garden and field monitoring (Figures 2 and 3; Table S1): plants producing floral morph‐1 (sampled from fruitful population of Maze and Pont) all were fruitful and seedful, while plants producing floral morph‐2 (sampled from fruitless populations of Orle, Poui, Gill, Chât, and Cham) remained fruitless and seedless. The flowers of fruitless populations became dehiscent 3 days after opening (Figure 2a,b).

**FIGURE 5:**
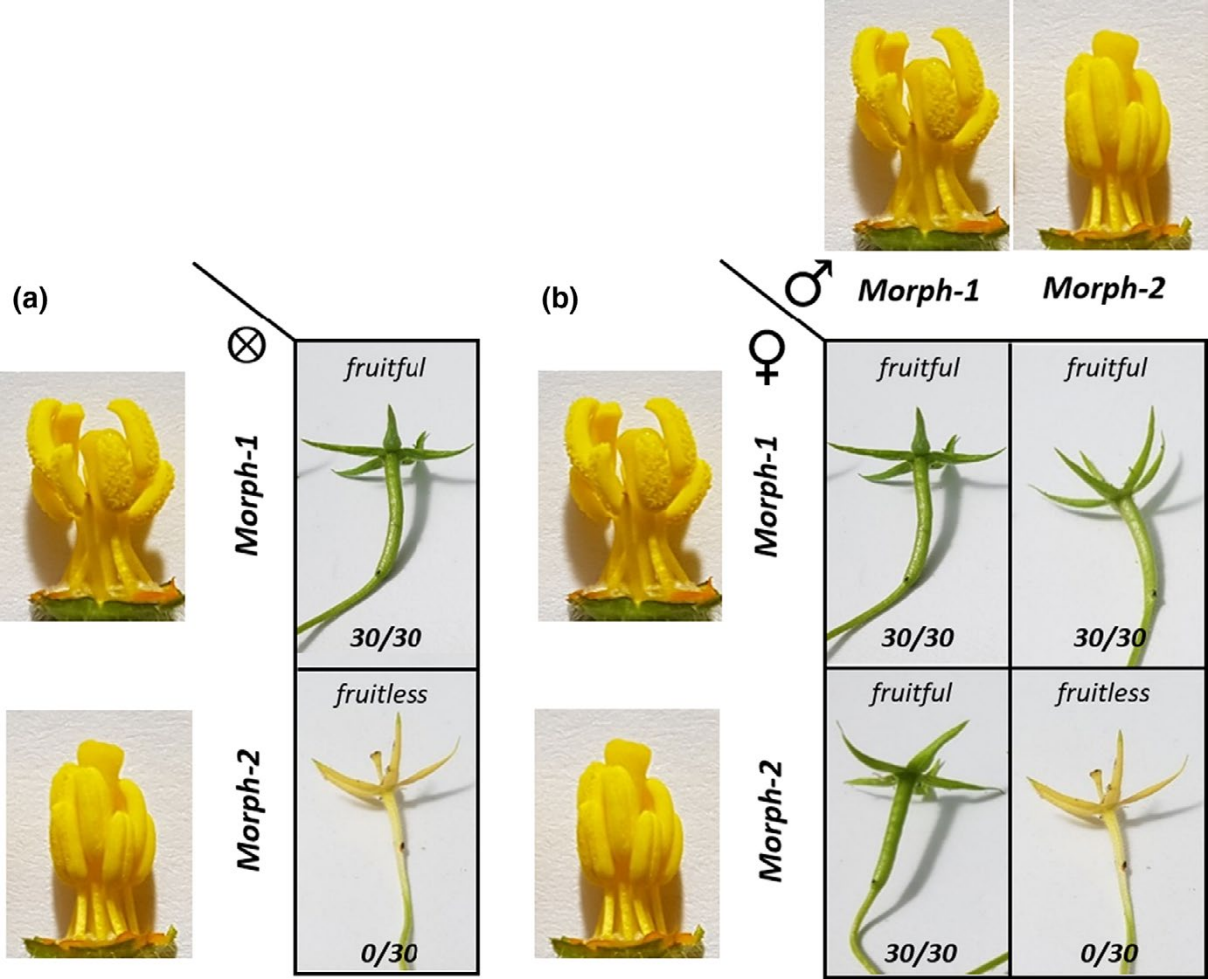
Fruit‐set in function of floral morphs and types of crosses in *Ludwigia grandiflora* subsp *hexapetala*. (a) Result of self‐pollination of morph‐1 or morph‐2. (b) Result of intra‐morph and reciprocal inter‐morph crosses between morph‐1 (fruitful populations) and morph‐2 (fruitless populations). Green fruit were fruits in formation with developing seeds (fruitful); yellow flowers were dehiscent flowers with no fruit formation and no seeds (fruitless). Numbers separated by a slash indicate the ratio between fruits obtained from a fixed—30—number of flowers. In self‐pollination and intra‐morph cross‐pollination, only morph‐1 populations produced fruits. In inter‐morph cross‐pollination, all crosses with morph‐1 used as male or female gave fruit

When we supplied morph‐1 flowers of plants sampled from fruitful populations with pollen from morph‐2 flowers from plants sampled from fruitless populations, they still all produced fruits and seeds. However, when we supplied morph‐2 flowers of plants sampled from fruitless populations with pollen of morph‐1 flowers from plants sampled from fruitful populations, they all were fruitful and seedful (Figure 2a,b). Repeating hand‐controlled pollination at seven temperatures (18°C, 22°C, 27°C, 30°C, 35°C, 40°C, and 45°C) in the greenhouse always gave the same results: morph‐2 flowers remained fruitless when self‐pollinated and pollinated with pollen from other morph‐2 flowers but became fruitful when supplied with pollen from morph‐1 flowers. Morph‐1 flowers all produced fruits and seeds whatever the provenance of the pollen (Figure 5). Fruit produced from morph‐1 and morph‐2 crosses contained similar seed‐set and germination rate (>87%) (Figures S5 and S6).

### 3.5 Fruit set in other worldwide native and invasive populations

Photos collected on the web of native and invasive populations confirmed the existence of two morphs using our floral morphometry in other worldwide populations (Table S3). Historical and recent data showed the presence of both floral morphs in the native area of *Lgh* (Argentina, Southern Brazil, Uruguay) as well as in invaded areas (North America, Europe). Populations in which we only detected morph‐2 flowers from collected photos were always congruent with populations being reported fruitless while populations with morph‐1 flowers only were all reported as being fruitful. Interestingly, invasive populations with only morph‐2 flowers were more frequently reported in web databases (11 times out of 18) than in its native area (four times out of 13; binomial probability).

## 4 DISCUSSION

Our results argued that the geographical distribution of a self‐incompatible morph, rather than biotic or abiotic environmental conditions, explained the success of the sexual reproduction of invasive populations of *Lgh* in Western Europe. Our results also argued for the first evidence of a self‐incompatibility system coinciding with two different floral morphs, with this worldwide invasive species having one short‐styled morph (corresponding to morph‐1) and one long‐styled morph (corresponding to morph‐2). If it were to be confirmed by additional studies, our results would constitute the first evidence for a heteromorphic self‐incompatibility system in the Ludwigia genus and the Onagraceae family.

### 4.1 Environmental variations in Western Europe did not explain fruitless populations of *L. grandiflora* subsp. *hexapetala*

Temperature was believed to be the main factor affecting *Lgh* fruitfulness and fertility (Dandelot et al., 2005). Indeed, increases or decreases in temperature after flower induction in some other plant species may deteriorate the production of viable male gametes and cause male sterility (Liu et al., 2019; Santiago & Sharkey, 2019). In France, fruitful populations were initially observed in the Atlantic zone, while fruitless populations were found in the Mediterranean zone, leading to the hypothesis that climate affected the reproductive success of *Lgh* (Dandelot et al., 2005). Our study, which focused on the Loire basin, confirmed the presence of fruitful populations in the Atlantic area, but also showed the presence of fruitless populations in this area. Fruitful populations were found in places with lower mean summer temperatures and lower mean summer rainfall. However, plants sampled from fruitless populations remain fruitless and seedless in common environmental conditions, in a common garden, or when reproducing in the greenhouse between 18°C and 45°C. In these same experimental conditions, all plants sampled from fruitful populations were fruitful, seedful and gave viable seeds, as observed in field populations. These results reject the hypothesis that the temperature and all other abiotic and biotic environmental variations (e.g., soils, pollinator cohorts, etc.) in the sampled places explain fruitless populations.

### 4.2 First evidence of a heteromorphic self‐incompatibility system found in *L. grandiflora* subsp. *hexapetala*

Our hand‐controlled cross‐pollinations showed that *Lgh* presented both self‐compatible and self‐incompatible populations in Western Europe. When studying populations in Western France, Haury et al. (2012) reported few cases of fruitless invasive populations becoming fruitful for the first time. After evidencing the self‐incompatibility system coinciding with two floral morphs, we more recently found that five of our monitored (Saint‐Avertin, Saint‐Aignan‐sur‐cher, Azay‐sur‐cher, Saint‐Aignan‐couflon, and Le‐port) but not sampled for common garden experimentations in situ populations observed to be fruitful resulted in fact in a mixture of individuals producing morph‐1 flowers and individuals producing morph‐2 flowers. In early October, all individuals in these populations produced fruits regardless of their floral morph, under free pollination. The coexistence of one compatible and one self‐incompatible type in the same invasive front raises questions concerning the maintenance of the self‐incompatible morph, and the evolution of the floral morphs in the next generations (De Cauwer et al., 2020), as well as their impacts on the invasibility of the species (Petanidou et al., 2012). Does the self‐incompatible morph provide some ecological or genetic advantage so that it is maintained on an invasive front? The fact that the self‐incompatible morph‐L was slightly more frequently reported in invasive populations than in its native area tackles the Baker's rule (Baker, 1955), and more recent results, for example those obtained for *Echium plantagineum* and *Solanum elaeagnifolium* (Petanidou et al., 2012) two other self‐incompatible species where the self‐compatible lines show an advantage over self‐incompatible in colonizing new areas. It highlights the necessity to carry out improved, more in‐depth investigations into the sexual reproductive system of *Lgh*. The first step will be to confirm and characterize the type of incompatibility system by tracking pollen germination and ovule fertility in both morphs, as has been done for *Guettarda speciosa* (Xu et al., 2018) and *Primula oreodoxa* (Yuan et al., 2018).

Raven (1979) studied the mating system in Onagraceae and classified the breeding systems of all 674 species: 283 (42%) are classified as outcrossing; 353 (52%) as self‐pollinating, and less than 6% (38) have a mixed breeding system. Among the 80 species of Ludwigia genera, 26, 54, and 0 species were classified as outcrossing, self‐pollinating, and mixed breeding systems, respectively. Our results revealed that *Lgh* was not only strict allogamous but also reproduced using a mixed mating system relying on a self‐incompatible system coinciding with two floral morphs. Our identification of a self‐incompatible floral morph and a self‐compatible floral morph in *Lgh* thus calls for a reappraisal of self‐incompatible systems in other *Ludwigia* spp. and Onagraceae in general.

### 4.3 Floral morphs are associated with sexual reproductive success in *Ludwigia grandiflora* subsp. *hexapetala*

We found that fruitful and fruitless populations of *Lgh* showed different floral morphologies and merosity. In the *Ludwigia* genus, variations in merosity have already been reported between species (Wagner et al., 2007). Here, for the first time in the *Ludwigia* genus, we showed that merosity variations occurred between and within populations of a single species, *Lgh*, and its distribution may be linked to its floral morphs. It questions the ecological and evolutionary importance of such biological features in this genus. Is this diversity in floral morphology maintained due to interactions with pollinators (Fenster et al., 2004), some negative‐frequency advantage associated with heterostyly (Barrett, 2019), or is it due to some specific biological constraint in this species? Beyond merosity, the analysis of floral morphometry highlighted the existence of two reciprocal herkogam morphs whose mating types reciprocally differ in stigma and anther height, with i/ a stamen–pistil ratio greater than 1.25 and of less than 0.9, and ii/ the pistils of the morph‐S flowers always 1–2 mm smaller than the pistils of the morph‐L, matching criteria for functional herkogamy, and criteria for a heteromorphic system, respectively (Barrett, 2019). The floral characteristics we found in *Lgh* corresponded to two other well‐known distylous species: *Fagopyrum esculentum* (Li et al., 2017) and *Linum suffruticosum* (Ruiz‐Martín et al., 2018). Both of these species show a non‐tubular dystilous flower structure as we also found in *Lgh* (Figure S7).

An old and abundant literature discussed floral morphology in Onanagracea, in particular for the *Ludwigia* species (Eyde, 1977; Raven, 1979). But, to our knowledge, floral dimorphism has been never mentioned in this family and genera before. We suppose that the reason for this omission could be that the morphological criteria were too subtle to be distinguished by eye and without dedicated measures. In addition to floral morphology, the main functional characteristic of heteromorphic systems is their assortative incompatibility, implying that all morphs are expected to be self‐ and intra‐morph incompatible (Barrett, 2019). However, several species have already been listed where one of the morphs is self‐compatible, or the two morphs show different rates of self‐compatibility (Brys & Jacquemyn, 2009; Ganguly & Barua, 2020; Ornduff, 1988; Zhou et al., 2015). For example, this is the case for the distylous *Luculia pinceana*, which presents a self‐compatible long‐styled morph and a self‐incompatible short‐styled morph, while both morphs are intra‐morph compatible (Zhou et al., 2015).

Mapping the biogeography of fruitful and fruitless populations of *Lgh* in native and invaded areas with self‐incompatible and ‐compatible morphs would help future studies in terms of the understanding of genetic diversity, ecology, and evolution of this species, and will allow us to trace the timing and the routes of its spread worldwide, identifying vectors, and characteristics of favorable environments.

### 4.4 Self‐ and inter‐morph compatible system calls for increased management efforts on fruitful populations

*Lgh* is known as one of the most threatening invasive freshwater plants worldwide. Its wide range of environmental tolerance in terms of fruitfulness and fertility may partly explain its worldwide invasiveness, although managers should not uniquely consider environmental conditions or climate changes when trying to limit its expansion and proliferation. Modeling of *Lgh* dispersal in terms of the climate predicts that its spread should increase up to twofold in Europe and North America (Gillard et al., 2017). Suitable new areas will mainly be located to the north of its current range.

However, we showed that seed set and fruit set were not affected by temperature in Western Europe. Sexual reproduction in those areas may exacerbate its expansion and proliferation, and should be considered in future plans for worldwide control. Indeed, it is only a matter of time before fruitless populations meet an incoming compatible morph and thus become fruitful. Sexual reproduction in *Lgh* may decisively participate in its dispersal and thereby increase its invasiveness. Indeed, floating seeds present a greater dispersal distance than clonal fragments: over 1000 km using water flow (Ruaux et al., 2009) and transport by vertebrates (García‐Álvarez et al., 2015). The presence and persistence of *Lgh* sexual seeds in seed banks (Grewell et al., 2019) highlight the importance of considering sexual reproduction in the resilience of this species when devising management plans. *Lgh* develops in invaded areas as dense mats with a mean of 77 stems par m^2^ (own in situ observations). Using our fruit‐set and seed‐set measures; this will result in a seed production of roughly 50,000 seeds per m^2^ of dense mat (77 stems/m^2^ × 11.5 fruits/stem × 60 seeds/fruit = 53130 seeds). Current management plans for invaded areas mainly rely on clonal propagation (Dandelot, 2004). The discovery that a temporal lack of compatible pollen suspends seed production is a definite game changer in terms of strategies defined to control this species. In contradiction with Baker's conjecture (Pannell, 2015), populations at the forefront of the invasion in the populations we monitored in the Loire basin, and in the worldwide database we analyzed (Europe, North America and Asia), were more frequently due to morph‐L, the self‐incompatible morph. This may be due to either an ecological advantage for settling vanguard invasive populations, or a reproductive strategy with investment in clonal propagation rather than in sexual reproduction. On top of this, sexual reproduction with massive recombination can also generate new abilities and favor local adaptations through new genetic and epigenetic combinations, which can then be maintained locally through clonal reproduction. Interestingly, one of the first populations able to reproduce sexually area newly present unusual adaptation to the terrestrial environment through genetic and epigenetic factors (Billet et al., 2018; Genitoni et al., 2020). To limit the risk of the appearance and dispersal of new genotypes, and indirectly to avoid a secondary invasion, management recommendations should pay particular attention to fruitful populations, and regulate seed production, for example by preferentially planning elimination actions at the beginning of blooming to limit fruit and seed production.

In conclusion, we rebutted the claims that environmental conditions limited sexual reproduction in invasive populations of *Lgh*, as was conjectured by previous literature and management plans (Dandelot, 2004). We also reported the first evidence of a heteromorphic incompatible system, with a self‐ and inter‐incompatible morph‐L and a self‐intra‐ and inter‐compatible morph‐S in invasive populations of *Lgh* in Western Europe. It would constitute the first evidence of this SI system in Onagraceae. An improved characterization of its heteromorphic incompatibility system in its physiological mechanism, and its genetics, should help us to understand its ecology and evolution, especially in invaded areas, and thereby be used to rationalize management plans.

## CONFLICT OF INTEREST

The authors declare no conflict of interest.

[Correction added on 18 June 2021, after first online publication: Conflict of Interest statement added to provide full transparency.]

## AUTHOR CONTRIBUTION

LP and DB designed this project. MB, MH, LP, and DB performed all experiments. JC, JH, and SS participated capsule husking. LP, SS, and DB analyzed data and wrote the manuscript. All authors read the manuscript.

## Supporting information

Supplementary Material

## Data Availability

Upon acceptance of this manuscript, all data will be uploaded in dryad and made available to the public.
