# Supplementary material for: Self‐incompatibility limits sexual reproduction rather than environmental conditions in an invasive water primrose
Source: Plant Environ Interact. 2021 Mar 29;2(2):74–86. doi: 10.1002/pei3.10042 (PMC10168087; doi:10.1002/pei3.10042)
Supplement: Supplementary file 1 — Supplementary Material [file PEI3-2-74-s001.docx]

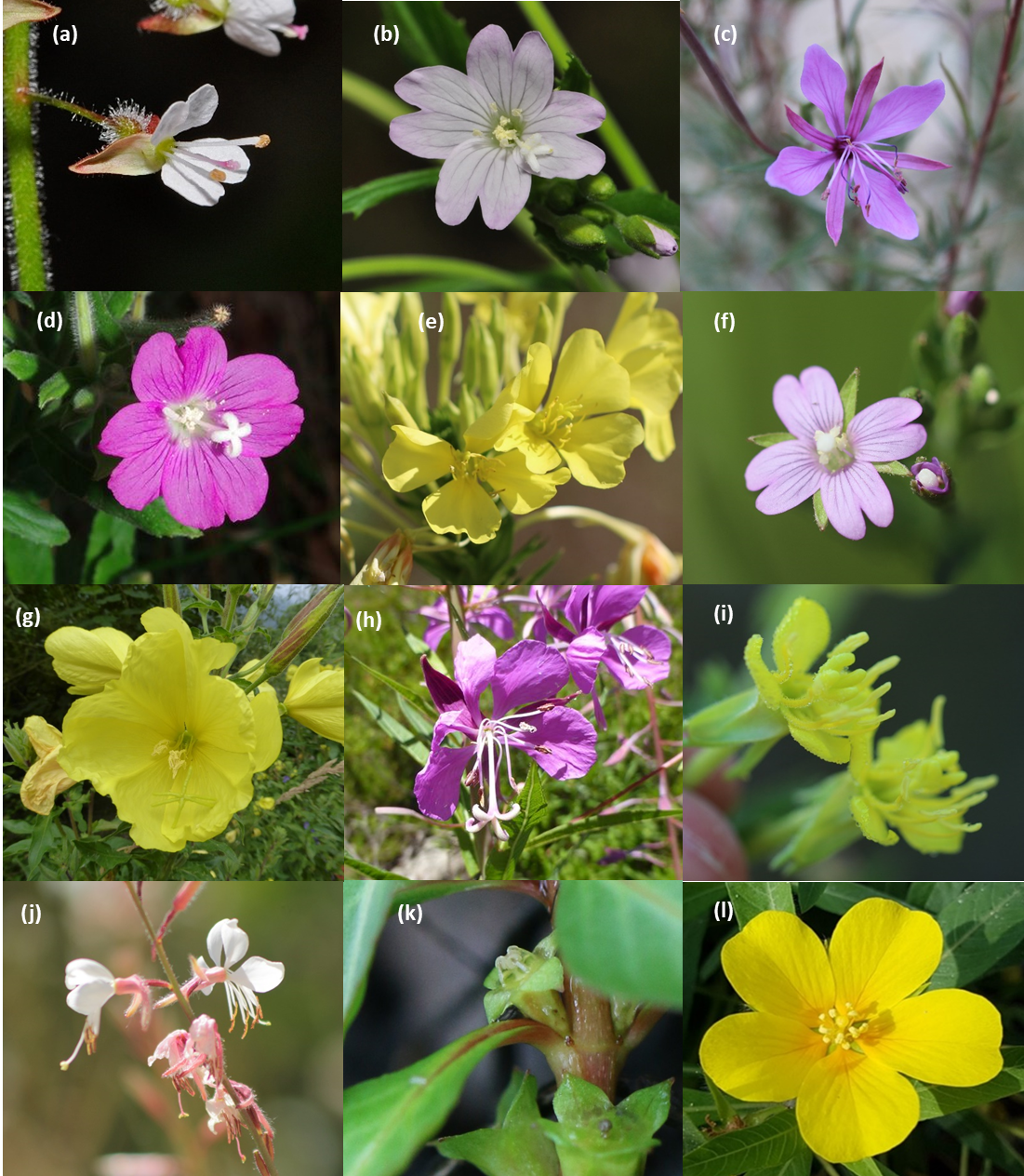
**Supporting Information**

**Figure S1:** Floral diversity in Onagraceae family**.** (a) *Circaea lutetiana*. (b) *Epilobium montanum.* (c) *Epilobium dodonaei.* (d) *Epilobium hirsutum.* (e) *Oenothera pycnocarpa.* (f) *Epilobium tetragonum.* (g) *Oenothera glazioviana.* (h) *Chamerion angustifolium.* (i) *Oenothera subterminalis.* (j) *Gaura longiflora.* (k) *Ludwigia palustris.* (l) *Ludwigia grandiflora subsp hexapetala.* (a to k) images courtesy of Romain Deschamps, (l) image courtesy Luis Portillo.


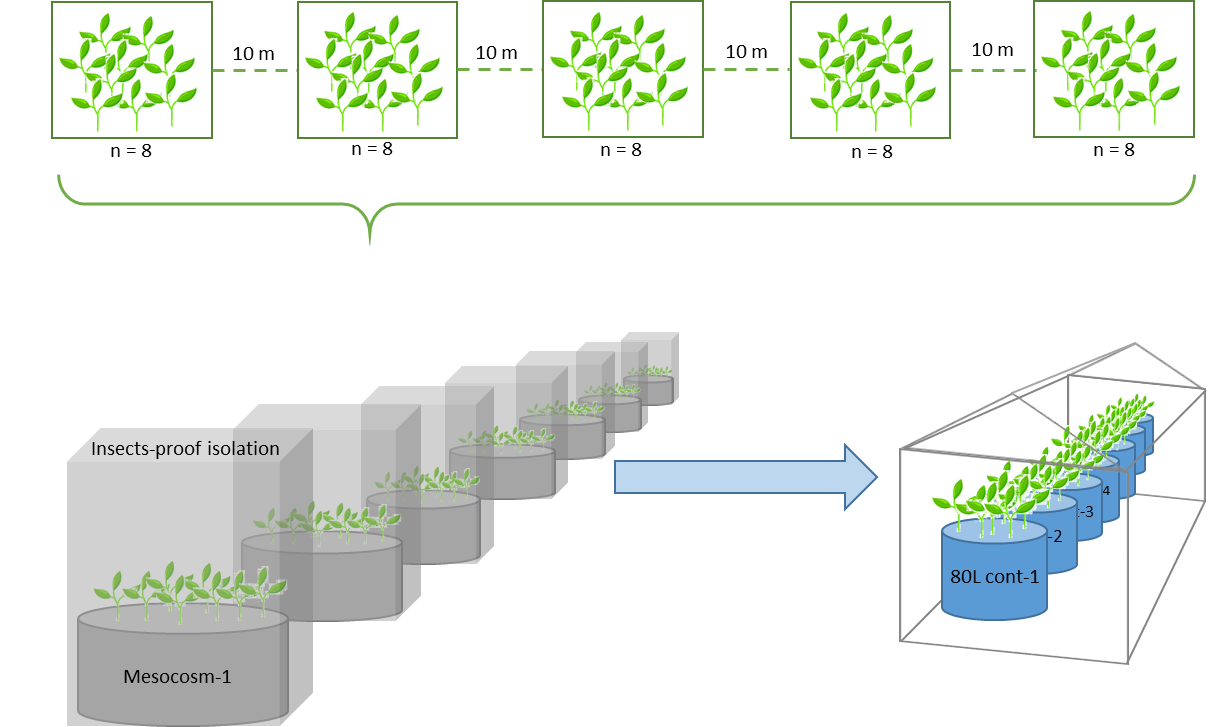


40 cloned-individuals

Installed in 450L mesocosm

**Common garden & Greenhouse**

***In situ* *Ludwigia grandiflora* subsp *hexapetala* sampled populations**

10 individuals subsampled

Installed in 80L container

**Figure S2:** Sampling and experimental setup of *Ludwigia grandiflora* subsp. *hexapetala* populations *in situ*, in common garden and greenhouse conditions. In the common garden, all 40 individuals sampled from populations bred together in a same mesocosm. In the greenhouse, all subsampled individuals were bred together in the same container.


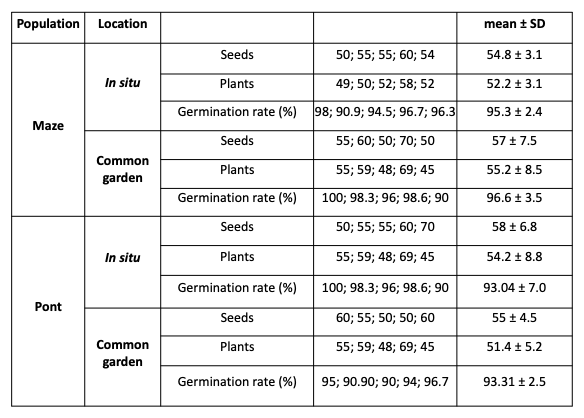


**Figure S3:** Seed-set, plant production and germination rate (%) in both fruitful populations of *Ludwigia grandiflora* subsp. *hexapetala in situ* and in common garden (Mazerolles: Maze; Pont-de-Cé: Pont).

Seeds: Seed-set from 5 randomly selected fruits produced *in situ* or in common garden; Plants: Plant production from seeds of 5 randomly fruits produced *in situ* or in common garden. Germination rate was calculated as the ratio of plant production over seed-set per fruit and was expressed in percentage. Fruits produced from both fruitful populations contained similar seed-set and plant production. Seed-set from Mazerolles and Pont-de-Cé showed similar germination rate (>93%).


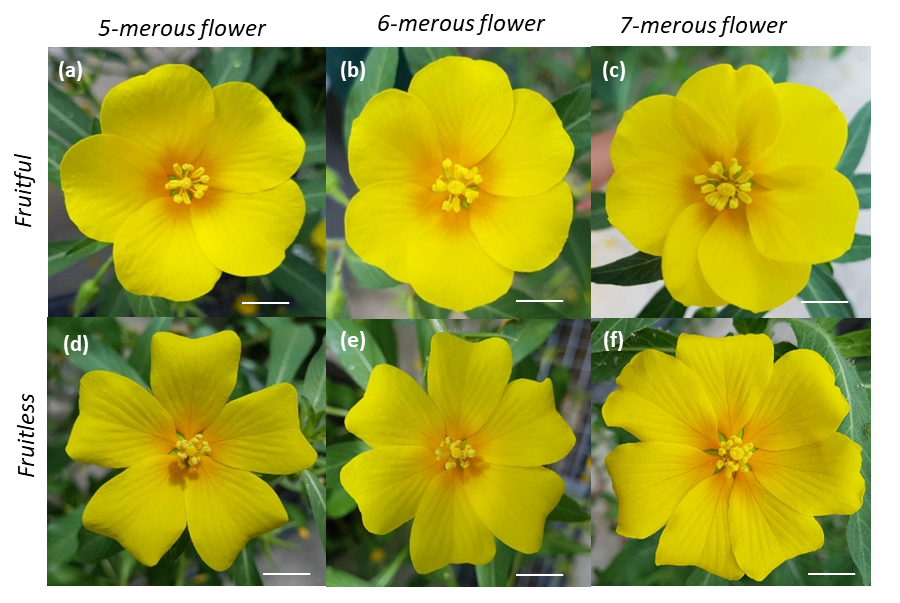


**Figure S4:** Floral morphology variation of *Ludwigia grandiflora* subsp *hexapetala* (2n=80): (a,b,c) 5-, 6- and 7-merous flowers from fruitful populations. (d,e,f) 5-, 6- and 7-merous flowers from fruitless populations. Floral formula for 5-merous flowers (a,d) were ⚥, Bt2, K5 + C5 + A10 + G(5); for 6-merous flowers (b,e) were ⚥, Bt2, K6 + C6 + A12 + G(6) and 7-merous flowers (c,f) were ⚥, Bt2, K7 + C7 + A14 + G(7). Bars = 1cm.


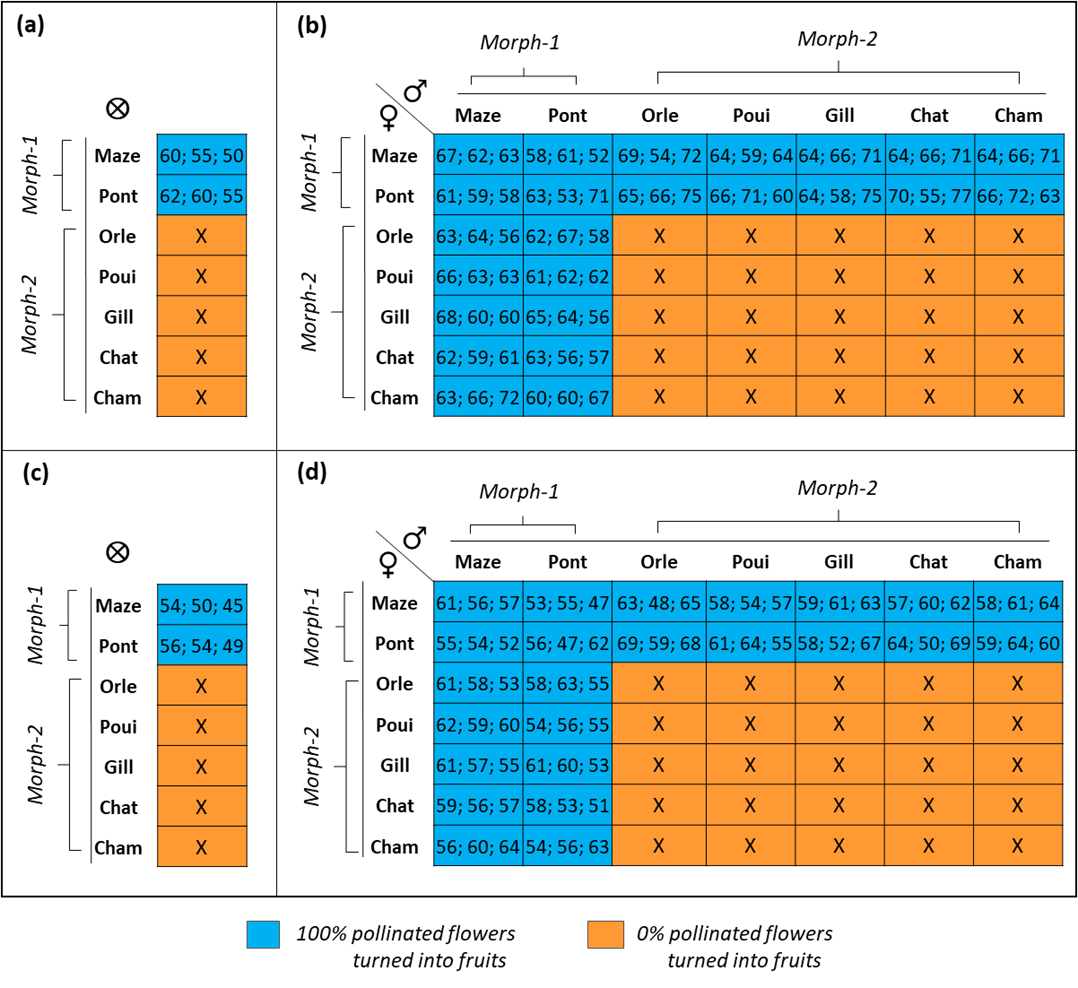
**Figure S5:** Fertility in the 7 sampled populations of *Ludwigia grandiflora* subsp. *hexapetala* after hand-controlled-pollination crosses from mid-July to beginning of August 2018 in greenhouse. (a) Seed-set from 3 randomly selected fruits produced by self-pollination. (b) Seed-set from 3 randomly selected fruits produced by cross-pollinations. (c) Plant production from 3 randomly fruits produced by self-pollination. (d) Plant production from 3 randomly selected fruits produced by cross-pollination. Numbers separated by semicolons stands for the number of seeds (a, b) and fully-developed plant (c, d) obtained from fruit1, fruit2, and fruit3. Cross sign indicates no seed or no plant obtained. Fruit produced from morph-1 and morph-2 crosses contained similar seed-set and plant production.

**Figure S6:** Germination rate (%) in the 7 sampled populations of *Ludwigia grandiflora* subsp. *hexapetala* after hand-controlled-pollination crosses from mid-July to beginning of August 2018 in greenhouse.

(a) Germination rate (%) from seed-set of 3 randomly selected fruits produced by self-pollination in morph-1. (b) Germination rate (%) from seed-sets of 3 randomly selected fruits produced by cross-pollination (Morph-1 x Morph1; Morph-1 x Morph-2; Morph-2 x Morph-1). Cross sign indicates no seed-set obtained. Germination rate was calculated as the ratio of plant production over seed-set per fruit and was expressed in percentage. Seed-set produced from morph-1 and morph-2 crosses showed similar germination rate (>87%).

**
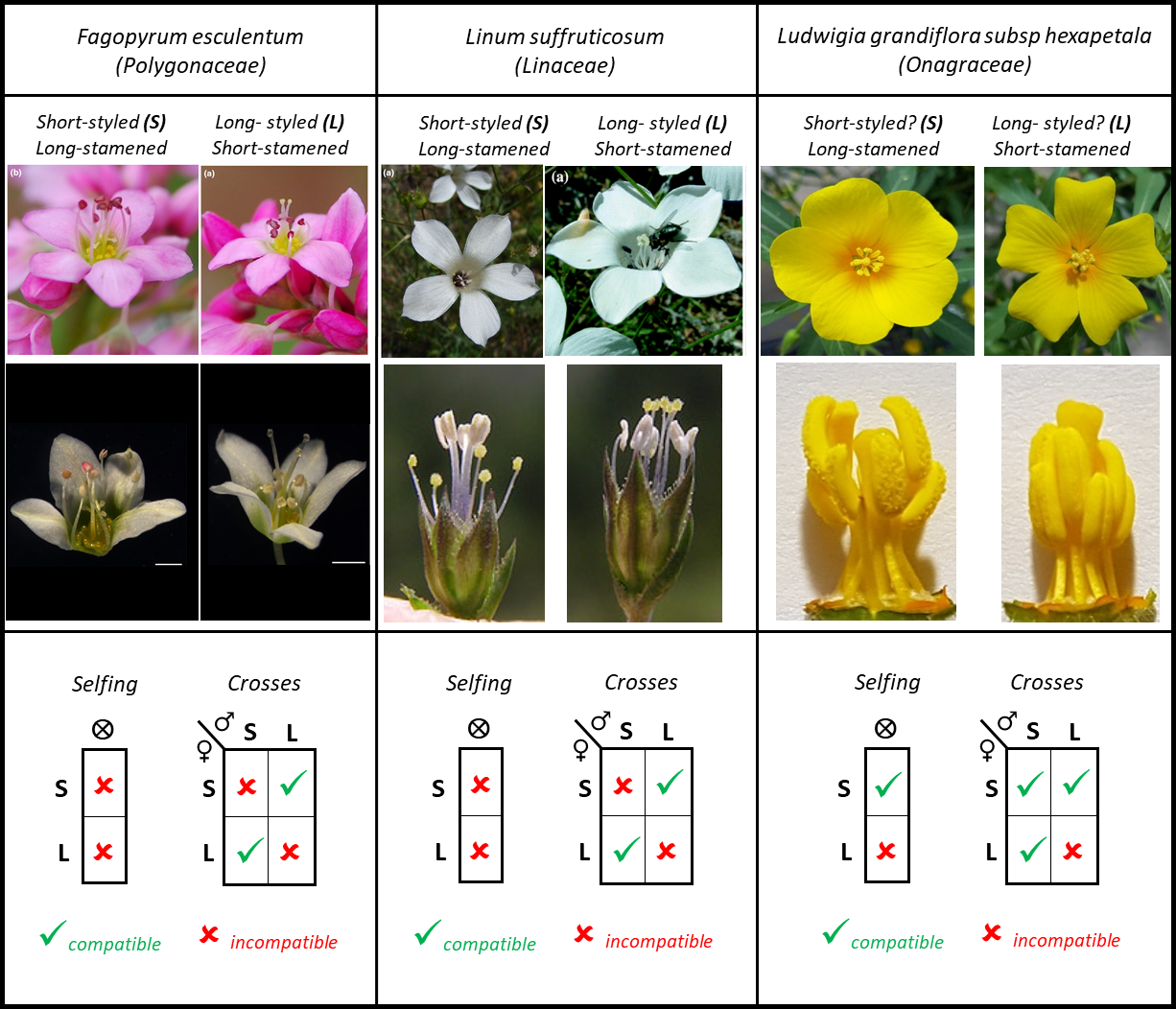
**

**Figure S7**: Floral morphology and reproductive system (compatible and incompatible cross) of 3 heteromorphic species: Left: *Fagopyrum esculentum* (pink flower from Barret *et al.* 2019; white flower from Li *et al*. 2017). Middle: *Linum suffruticosum* (*Barret et al.* 2019, Ruiz-Martín *et al.* 2018) Right: *Ludwigia grandiflora* subsp. *hexapetala* and mating schemas by Luis Portillo.

“S” stands for short-styled flower, “L” for Long-styled flower. Green checked signs indicate fruitful and fertile crosses while red crosses indicate fruitless and infertile crosses.

**Table S1:** Locations of all studied fruitful and fruitless populations in the Loire basin. The first part of table concerned seven sampled populations and the other populations were classified from East to West in Loire-Bretagne basin.

| **Number** | **Abbreviation** | **Population** | **GPS Location** | | **Fruitfulness** |
| --- | --- | --- | --- | --- | --- |
| 7 | Cham | Chambéon | 45°41'03.4"N | 4°12'12.1"E | Fruitless |
| 6 | Chat | Châtel-de-Neuvre | 46°24'05.4"N | 3°19'10.1"E | Fruitless |
| 5 | Gill | Gilly-sur-Loire | 46°31'40.6"N | 3°48'25.7"E | Fruitless |
| 4 | Poui | Pouilly-sur-Loire | 47°16'48.4"N | 2°57'25.2"E | Fruitless |
| 3 | Orle | Orléans | 47°53'41.3"N | 1°55'48.6"E | Fruitless |
| 2 | Pont | Pont-de-Cé | 47°25'40.7"N | 0°31'28.9"W | Fruitful |
| 1 | Maze | Mazerolles | 47°23'17.3"N | 1°28'07.4"W | Fruitful |
|  |  | Roanne | 46°03'48.6"N | 4°06'53.0"E | Fruitless |
|  |  | Cournon-d’Auvergne | 45°43'59.1"N | 3°12'53.4"E | Fruitless |
|  |  | Monetay-sur-Allier | 46°22'55.1"N | 3°18'28.1"E | Fruitless |
|  |  | Vichy | 45°43'59.1"N | 3°12'53.4"E | Fruitless |
|  |  | Digoin | 46°28'48.3"N | 3°58'24.7"E | Fruitless |
|  |  | Rigny-sur-Arroux | 46°32'02.1"N | 4°01'56.3"E | Fruitless |
|  |  | Nevers | 46°59'06.5"N | 3°09'36.0"E | Fruitless |
|  |  | Gien | 47°41'25.6"N | 2°36'32.9"E | Fruitless |
|  |  | Sully-sur-Loire | 47°46'28.4"N | 2°23'07.7"E | Fruitless |
|  |  | Blois | 47°35'13.3"N | 1°20'16.7"E | Fruitless |
|  |  | Vierzon | 47°13'10.0"N | 2°03'26.8"E | Fruitless |
|  |  | Saint-Aignan-sur-Cher | 47°16'19.8"N | 1°22'32.1"E | Fruitless |
|  |  | Azay-sur-cher | 47°21'08.2"N | 0°51'01.2"E | Fruitful |
|  |  | Le Port | 47°21'38.2"N | 0°48'20.8"E | Fruitful |
|  |  | île-du-Chapeau-bas | 47°24'05.8"N | 0°52'18.2"E | Fruitless |
|  |  | Port-de-Vallières | 47°23'09.5"N | 0°36'31.0"E | Fruitless |
|  |  | Villandry | 47°20'30.3"N | 0°28'52.8"E | Fruitful |
|  |  | Montsoreau | 47°13'01.9"N | 0°03'25.2"E | Fruitful |
|  |  | Lac-de-Maine | 47°27'34.7"N | 0°35'30.8"W | Fruitful |
|  |  | Marais-du-Syl | 47°17'35.9"N | 1°56'17.0"W | Fruitless |
|  |  | Champs-d'Or | 47°19'31.1"N | 2°16'03.9"W | Fruitless |
|  |  | Sabot-d'Or | 47°19'14.1"N | 2°15'24.4"W | Fruitful |
|  |  | Brière-est | 47°22'05.8"N | 2°12'08.1"W | Fruitless |
|  |  | Isac-Vilaine | 47°34'38.5"N | 2°06'03.2"W | Fruitless |
|  |  | Marais-de-l'Isac | 47°33'38.1"N | 2°01'05.2"W | Fruitless |
|  |  | Apigné | 48°05'35.4"N | 1°44'25.6"W | Fruitless |
|  |  | Sarzeau | 47°30'43.5"N | 2°43'40.2"W | Fruitful |
|  |  | Léguer | 48°29'23.5"N | 3°20'29.6"W | Fruitless |
|  |  | Lac-de-Lébisay | 49°13'10.3"N | 0°21'07.4"W | Fruitless |
|  |  | Putanges-le-Lac | 48°45'49.6"N | 0°15'11.4"W | Fruitless |

**Table S2:** Mean values of floral parts morphometry data for fruitful and fruitless populations floral group 2 with their respective standard deviations (t-test, 0.05).

| **Floral parts** | **Floral fruitful population** | **Floral fruitless population** |
| --- | --- | --- |
| Sepal length (mm) | 18.33 ± 1.12 | 14.91 ± 1.18 |
| Sepal width (mm) | 4.20 ± 0.36 | 3.28 ± 0.29 |
| Petal length (mm) | 27.15 ± 2.21 | 21.00 ± 1.91 |
| Petal width (mm) | 22.42 ± 2.06 | 16.26 ± 1.51 |
| First whorl stamen length (mm) | 6.97 ± 0.63 | 6.31 ± 0.47 |
| Second whorl stamen length (mm) | 9.31 ± 0.49 | 7.59 ± 0.43 |
| First whorl anther length (mm) | 4.12 ± 0.37 | 3.24 ± 0.44 |
| Second whorl anther length (mm) | 4.14 ± 0.41 | 3.68 ± 0.47 |
| Floral receptacle width (mm) | 4.87 ± 0.42 | 3.74 ± 0.2 |
| Pistil length (mm) | 7.82 ± 0.35 | 8.31 ± 0.35 |
| Nectar production (µL) | 9.43 ± 1.59 | 4.93 ± 1.99 |

**Table S3:** Assignment of floral morphs over the world using criteria defined for morphs observed in France, in *Ludwigia grandiflora* subsp*. hexapetala* (Syn. *Ludwigia hexapetala*) populations. Considering the binary answer we obtained in our results, we qualified fruitfulness in populations as **^PF^** when fruitfulness was directly observable from the available photos (PF) on which we saw either aborted fruits (fruitless) or fleshy developing fruit (fruitful) and **^TR^** using textual reports wrote by managers, naturalists and scientifics.

Remark:

1) the list below included only images corresponding to the true species having a geographic origin, and showing at least one visible or measurable criterion of our biometrics.

2) Please note that many websites, even official and governmental ones, present significant and unfortunately frequent taxonomic errors. They annotated as *Ludwigia grandiflora* subsp*. Hexapetala* photos belonging to another *Ludwigia* species that can be distinguished by the shape of the leaves, presence / absence of trichomes on all plant organs, shape and size (length / width ratio) of the sepals, morphology of the lower ovaries, shape and size (length / width ratio) of fruits, deciduous or persistent sepals on fruit. Some examples:

(i) <https://bentonswcd.org/plant/large-flowered-primrose-willow/> Here photos correspond to *Ludwigia bonariensis*.

(ii) <https://www.inaturalist.org/observations/41006687> Here photos correspond to *Ludwigia leptocarpa* ;

(iii) <https://www.inaturalist.org/observations/5994468> ; here photos correspond to *Ludwigia peploides* subs. *glabrescens.*

(iv) <https://www.inaturalist.org/observations/14123028> here photos correspond to hexaploid *Ludwigia grandiflora* subsp. *grandiflora* (syn. *Ludwigia grandiflora*).

| **Type of Data** | **Status** | **Continent** | **Country** | **Floral morph** | **Location** | **Criteria for assigning the floral morphotype** | **Fruitfulness** | **Data collector** | **Link** |
| --- | --- | --- | --- | --- | --- | --- | --- | --- | --- |
| recent data | Invasive | Europe | Spain | Morph-1 | Calatuña (Ebre) | Stamens above stigmas, size of petals and sepals | Fruitless^TR^ | Luis portillo | <https://dugi-doc.udg.edu/bitstream/handle/10256/17154/NewContributions.pdf?sequence=1> |
| recent data | Invasive |  | Italy | Morph-2 | Varano Borghi VA | Yellow abortive "Floral vestiges" | Fruitless^PF^ | Luciano Arcorace Inaturalist | <https://www.inaturalist.org/observations/31437283> |
| recent data | Invasive |  | Italy | Morph-2 | Lombardia | Stamens below stigmas/Yellow abortive "Floral vestiges" | Fruitless^PF, TR^ |  | [http://www.biodiversita.lombardia.it/sito/index.php?option=com_content&view=article&id=220:c03-ludwigia&catid=89&Itemid=843](https://www.pianteacquatiche.org/ludwigia_grandiflora.html) |
| recent data | NA |  | Finland | Morph-1 | Kaisaniemi Botanic Garden, Helsinki, 20150921 | Stamens above stigmas / fruit formation / petals form | Fruitful^PF^ | Raino Lampinen | <https://www.flickr.com/photos/rainol/30304705903> |
| recent data | Invasive |  | United Kingdom | Morph-1 | Breamore Marsh, in New Forest District, | Size of sepals, petals and fruit/stamens above stigmas | Fruitful^PF, TR^ | Trevor Renals | <http://www.rinse-europe.eu/assets/Uploads/partner-annexe-demonstration-creeping-water-primrose.pdf> |
| recent data | Invasive |  | The Netherlands | Morph-2 | Hampshire, Lateraalkanaal, Almelo, Twente, Overijssel | Stamens below stigmas/Yellow abortive "Floral vestiges" | Fruitless^PF, TR^ | NA | <https://www.agefotostock.com/age/en/Stock-Images/Rights-Managed/PNA-19389682> |
| recent data | Invasive |  | Germany | Morph-1 | River Leda | Size of sepals, petals and fruit/stamens above stigmas | Fruitful^PF, TR^ | Stefan Nehring | <http://www.aquaticinvasions.net/2011/AI_2011_6_1_Nehring_Kolthoff.pdf> |
| recent data | Invasive |  | Belgium | Morph-2 | Péruwelz, Callenelle, Pont de Wiers | Stamens below stigmas/Yellow abortive "Floral vestiges" | Fruitless^PF, TR^ | NA | <http://alienplantsbelgium.be/content/ludwigia-hexapetala> |
| recent data | Invasive | North america | USA (California) | Morph-1 | [Sonoma, Lake & Mendocino County Coast, CA, US](https://www.inaturalist.org/observations?place_id=78829) | overlapping petals/Stamens above stigmas | Fruitful^PF^ | Observation © lilredhen | <https://www.inaturalist.org/observations/30446589> |
| recent data | Invasive | North america | USA (California) | Morph-1 | [Riverfront Regional bioblitz extension, US](https://www.inaturalist.org/observations?place_id=112184) | Stamens above stigmas | Fruitful^PF^ | Observation © Di | <https://www.inaturalist.org/observations/1895250> |
| recent data | Invasive |  | USA (California) | Morph-2 | [Spring Lake Regional Park, US, CA](https://www.inaturalist.org/observations?place_id=95070) | Stamens below stigmas | Fruitless^PF^ | Observation © Gary Morgret | <https://www.inaturalist.org/observations/14066827> |
| recent data | Invasive |  | USA (California) | Morph-2 | [Golden Gate Natl. Rec. Area - MA, CA, US](https://www.inaturalist.org/observations?place_id=5218) | Yellow abortive "Floral vestiges" | Fruitless^PF^ | Observation © Daniel George | <https://www.inaturalist.org/observations/3966627> |
| recent data | Invasive |  | USA (California) | Morph-2 | [Yolo Bypass Wildlife Area, CA, US](https://www.inaturalist.org/observations?place_id=3878) | Stamens below stigmas | Fruitless^PF^ | Observation Par Justin | <https://www.inaturalist.org/observations/681894> |
| recent data | Invasive |  | USA (Virginia) | Morph-1 | [Appalachian Mountains, US](https://www.inaturalist.org/observations?place_id=124637) | Size of sepals, petals and fruit | Fruitful^PF^ | Observation © stinger | <https://www.inaturalist.org/observations/28469257> |
| recent data | Invasive |  | USA (North California) | Morph-2 | Griffith St, Davidson, N | Yellow abortive "Floral vestiges" | Fruitless^PF^ | Observation © souplala | <https://www.inaturalist.org/observations/28570505> |
| recent data | Invasive |  | USA (South California) | Morph-2 | Russellville | Stamens below stigmas | Fruitless^PF^ | Observation © BJ Stacey | <https://www.inaturalist.org/observations/13382910> |
| recent data | Invasive |  | Mexico | Morph-2 | [Sierra Cerro de la Silla ANP Estatal, NL, MX](https://www.inaturalist.org/observations?place_id=146901) | Stamens below stigmas/Yellow abortive "Floral vestiges" | Fruitless^PF^ | Observation © Pedro Alanis | <https://www.inaturalist.org/observations/11944252> |
| recent data | Invasive | Asia | Japan | Morph-2 | Lake Biwa | Stamens below stigmas/Yellow abortive "Floral vestiges" | Fruitless^PF, TR^ | Shinya Hieda | <https://www.jstage.jst.go.jp/article/apg/71/1/71_201911/_pdf/-char/ja> |
| recent data | Native | South America | Argentina | Morph-2 | Buenos Aires | Stamens below stigmas/Yellow abortive "Floral vestiges" | Fruitless^PF^ | Instituto de Botánica Darwinion | <http://www.darwin.edu.ar/Proyectos/FloraArgentina/DetalleEspecie.asp?forma=&variedad=&subespecie=hexapetala&especie=grandiflora&genero=Ludwigia&espcod=1776> |
| recent data | Native |  | Argentina | Morph-2 | Entre Ríos | Stamens below stigmas/Yellow abortive "Floral vestiges" | Fruitless^PF^ | Instituto de Botánica Darwinion | <http://www.darwin.edu.ar/Proyectos/FloraArgentina/DetalleEspecie.asp?forma=&variedad=&subespecie=hexapetala&especie=grandiflora&genero=Ludwigia&espcod=1776> |
| recent data | Native |  | Argentina | Morph-2 | Santa Fé | Stamens below stigmas/Yellow abortive "Floral vestiges" | Fruitless^PF^ | Instituto de Botánica Darwinion | <http://www.darwin.edu.ar/Proyectos/FloraArgentina/DetalleEspecie.asp?forma=&variedad=&subespecie=hexapetala&especie=grandiflora&genero=Ludwigia&espcod=1776> |
| recent data | Native | South America | Argentina | Morph-1 | Corrientes | Size of sepals, petals and fruit/stamens above stigmas | Fruitful^PF, TR^ | Instituto de Botánica Darwinion | <http://www.darwin.edu.ar/Proyectos/FloraArgentina/DetalleEspecie.asp?forma=&variedad=&subespecie=hexapetala&especie=grandiflora&genero=Ludwigia&espcod=1776> |
| recent data | Native |  | Argentina | Morph-2 | Río Negro | Stamens below stigmas/Yellow abortive "Floral vestiges" | Fruitless^TR^ | Instituto de Botánica Darwinion | <http://www.darwin.edu.ar/Proyectos/FloraArgentina/DetalleEspecie.asp?forma=&variedad=&subespecie=hexapetala&especie=grandiflora&genero=Ludwigia&espcod=1776> |
| recent data | Native |  | Argentina | Morph-1 | San Luis | Size of sepals, petals and fruit/stamens above stigmas | Fruitful^TR^ | Instituto de Botánica Darwinion | <http://www.darwin.edu.ar/Proyectos/FloraArgentina/DetalleEspecie.asp?forma=&variedad=&subespecie=hexapetala&especie=grandiflora&genero=Ludwigia&espcod=1776> |
| historical data | Native |  | Argentina | Morph-1 | Mendoza (1833) | Size of sepals, petals and fruit | Fruitful^PF, TR^ | Royal Botanic Garden Edinburgh | <https://data.rbge.org.uk/search/herbarium/?cfg=fulldetails.cfg&specimen_num=295482> |
| historical data | Native |  | Argentina | Morph-2 | Río Negro (1891) | Size of sepals and petals | Fruitless^PF, TR^ | Royal Botanic Garden Edinburgh | <https://data.rbge.org.uk/search/herbarium/?cfg=fulldetails.cfg&specimen_num=676929> |
| historical data | Native |  | Argentina | Morph-1 | Mendoza | Size of sepals and fruit | Fruitful^PF, TR^ | Kew Royal Botanical Garden | <https://apps.kew.org/herbcat/getImage.do?imageBarcode=K000533255> |
| historical data | Native |  | Argentina | Morph-2 | Patagonia (Río Negro ?) | Size of sepals and petals | Fruitless^PF^ | Kew Royal Botanical Garden | <https://apps.kew.org/herbcat/getImage.do?imageBarcode=K000533258> |
| historical data | Native |  | Argentina | Morph-2 | Buenos Aires | Size of sepals and petals | Fruitless^TR^ | Kew Royal Botanical Garden | <https://apps.kew.org/herbcat/getImage.do?imageBarcode=K000533257> |
| historical data | Native |  | Brazil | Morph-2 | Santa Catarina do Sul | Petals size | fruitless^TR^ | Kew Royal Botanical Garden | <https://apps.kew.org/herbcat/getImage.do?imageBarcode=K001077561> |
| historical data | Native |  | Uruguay | Morph-2 | NA | Size of sepals and petals | Fruitless^TR^ | Kew Royal Botanical Garden | <https://apps.kew.org/herbcat/getImage.do?imageBarcode=K000533256> |
